# The revolution will be open-source: how 3D bioprinting can change 3D cell culture

**DOI:** 10.18632/oncotarget.27099

**Published:** 2019-07-30

**Authors:** Robert D. Bruno, John Reid, Patrick C. Sachs

**Affiliations:** ^1^ School of Medical Diagnostic & Translational Sciences, Old Dominion University, Norfolk, VA, USA

**Keywords:** 3D bioprinting, 3D culture, mammary gland, breast cancer, open-source

The development of three-dimensional culture scaffolds represents a revolutionary step forward for in vitro culture systems. Various synthetic and naturally occurring substrates have been developed that support 3D growth of cells. In most fields, including mammary gland biology and tumorigenesis, the two most common substrates used are the basement membrane rich extracellur matrix (ECM) isolated from Engelbreth-Holm-Swarm (EHS) mouse sarcomas (e.g. Matrigel) and collagen extracted from rat-tails. The processes of 3D culture in these two substrates has remained unchanged for nearly half a century: cells are either mixed with unpolymerized matrix to disperse them randomly throughout the substrate upon polymerization or overlaid randomly on top of a preformed hydrogel. While effective in generating organoid/tumoroid structures, the random nature of these processes has many drawbacks that limit the reproducibility and tunability of the experimental design. Furthermore, random cellular distributions limit the utility of these substrates for studying interactions within the cellular microenvironment, which have been shown to be critical for the control of stem and cancer cell function [1].

To overcome these issues, computer numerically controlled (CNC) devices can be adapted to precisely control cellular deposition within hydrogels. An example of these devices can be found in the three-axis control of modern 3D fusion deposition method (FDM) printers. Despite a rapid drop in cost of 3D printing technology, printers specifically engineered for bioprinting purposes generally remain unattainably expensive for general biological research laboratories. Thus the technology has been limited to specific biofabrication applications in specialty biomedical engineering laboratories. Furthermore, commercially available bioprinters are exclusively designed for printing “bioinks” (unpolymerized scaffoldings with or without cells) into shapes. While potentially useful for medical reconstructive procedures, the shape of the hydrogel is meaningless to cell biologists seeking to understand basic questions of cell biology, or for engineering applications seeking to direct specific differentiation of cells.

**Figure 1:**
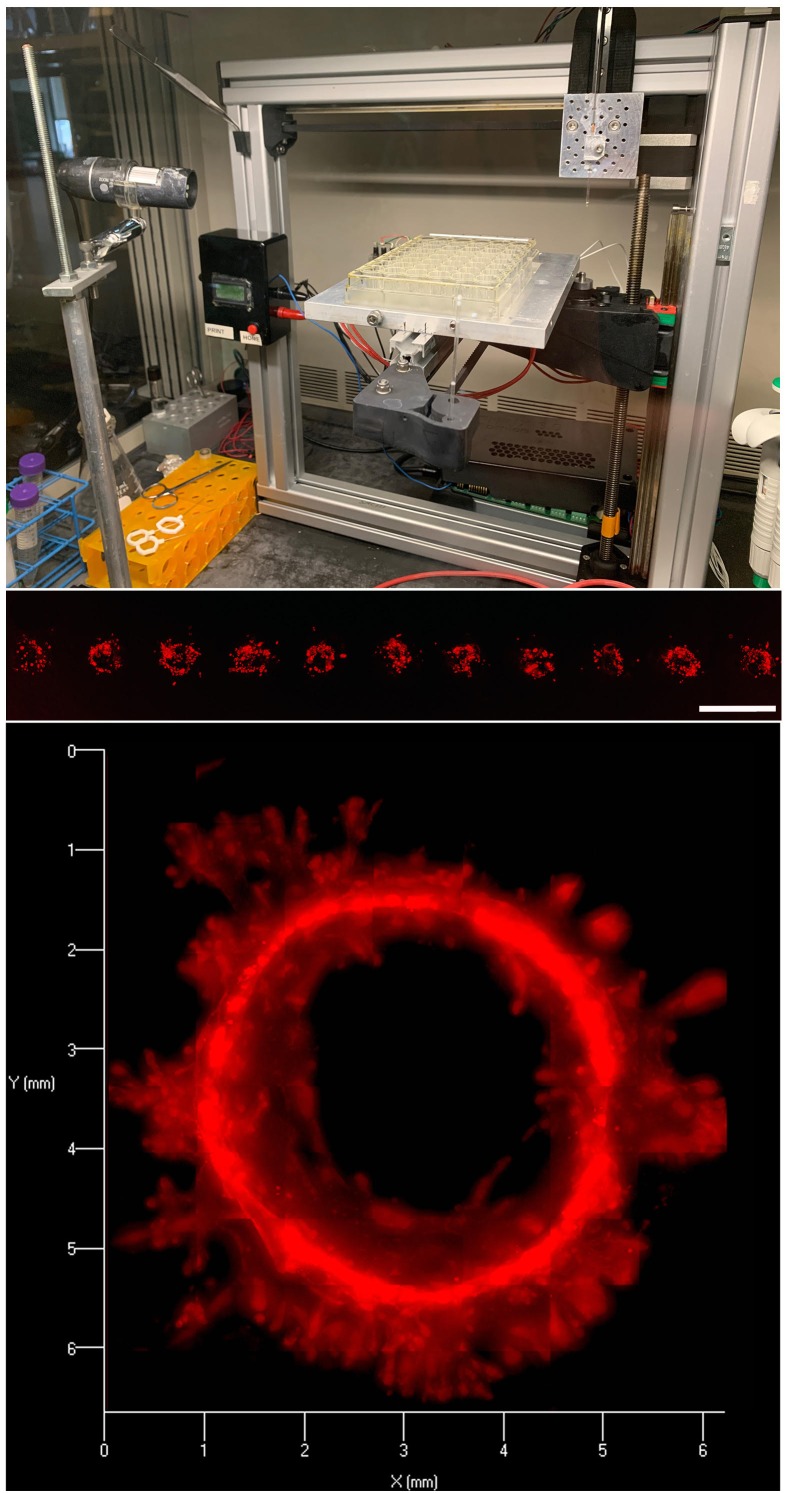
Custom benchtop 3D bioprinter and its application for printing large mammary organoids Top: Image of an example 3D bioprinter constructed off of the Felix 3.0 (FELIXrobotics, NL) platform. Middle: Example of coordinated print of clusters of red fluorescent protein (RFP) labeled MCF12a cells at distances of 200μm in linear array. Image taken 24 hours post-print. Scale bar = 200μm. Bottom: Mature organoid (21 days post-print) formed from coordinated print of clusters of RFP MCF12a cells into a circular array. Resulting organoids have been shown to have contiguous lumens stretching > 3mm in length.

To this end, we recently developed a low-cost open access 3D bioprinting system that can be used for scientists applications (Figure 1) [2]. The printer is an open source project that allows other laboratories to build their own system. Initially, all necessary parts can be printed using a standard off-the-shelf 3D FDM printer. The same printer can then be modified with these parts into a 3D bioprinter. In essence, you can 3D print your own 3D bioprinter. This system is designed to be adaptable to any application the end user requires, and we have described its use for both printing cells as well as guiding electrodes for directed electrical pulsing of cells [3, 4]. To increase precision and maintain integrity of printed cells, we use pulled glass micropipette syringes as our cell injection “print-head” [2]. Compared to standard steel needles attached to luer lock syringes, these glass micropipettes have a finer point and reduce sheer force on the cells. Combined, this minimizes disruptions to the cells and allows the hydrogel to seal behind the print. Thus, our system allows for the precise placement of cells, that then self-organize into organoids/tumoroids, making functional structures [4]. This differs from many bioprinting approaches that print cell free or cell-laden “bioinks” into 3D shapes. Again, while these approachees are potentially useful for specific medical reconstruction procedures, they have little utility for most biological applications. For example, cells printed in the shape of a mammary gland are not a mammary gland; it is the coordinated function and differentiation of cells through development that make tissues and organs functional.

We have recently described the use of this system for printing mammary organoids in standard 3D hydrogels [4]. By depositing specific numbers of cells at controllable distances we could guide the growth of organoids into predictable sizes and shapes (Figure 1). The key to the guided growth was the fact that mammary epithelial cells (MCF12a and MCF10a) would preferentially grow towards neighboring prints, forming single contiguous organoids. Using this strategy, we generated large contiguous luminal mammary organoids (> 5mm in length). This is in clear contrast to random culture where the dispersion of cells results in random organoid shape and size, with organoids never forming more than a couple of hundred microns in size. Controlling for organoid distancing, shaping, and sizing is thus not feasible in standard culture models and therefore interpretation of studies where these factors may play a role becomes difficult. This is particularly true for experiments on microenvironmental forces and cancer/epithelial cell growth. Because surrounding organoids can influence the rigidity of the microenvironment, control of the placement, spacing, and size is critical.

In an era of poor data reproducibility in science [5], instruments and methods designed to limit lab-to-lab variability are in need. The open source nature of our bioprinter helps facilitate standardization of experimental parameters across laboratories. This is because the machine instructions or GCODE generated for printing experiments can be shared once data is published. We have shared our files through our website http://www.odustemcell.org. Researchers can go to this website, download the files needed to print their own bioprinter, and then use the GCODE files necessary to repeat our experiments exactly using their own bioprinter. While this certainly doesn’t eliminate all inter-laboratory variability, it helps simplify the process of reproducing an experiment.

Our current focused application of this printing technology is to understand the role of the cellular microenvironment in controlling differentiation of stem and cancer cells (Reid et al., Submitted). We have explored this topic in in vivo models [6–10], but our bioprinting platform allows for mechanistic insights into the process. These studies are ongoing, but one can imagine our printing platform can be used to improve any application where the random nature of traditional 3D culture is a confounding variable. And, one could argue, nearly every study is potentially confounded by this factor.

A common issue with modern science/scientist is the tendency to assign radical ideas to the “science fiction” classification; however, bioprinting needs not be the stuff of science fiction. Our studies highlight the ease of access and the utility of the technology for basic cell and cancer biology studies. Thus, we hope to lower the bar of entry further by developing easier-to-access solutions, such as ready built kits, and a graphic user interface (GUI) to simplify the experimental programming. The system offers a potentially revolutionary step forward for 3D culture models of development and cancer.
